# Mutational profiling of low‐grade gliomas identifies prognosis and immunotherapy‐related biomarkers and tumour immune microenvironment characteristics

**DOI:** 10.1111/jcmm.16947

**Published:** 2021-10-01

**Authors:** Wen‐wen Lin, Guan‐yong Ou, Wei‐jiang Zhao

**Affiliations:** ^1^ Center for Neuroscience Shantou University Medical College Shantou Guangdong China; ^2^ Cell biology department, Wuxi School of Medicine Jiangnan University Wuxi, Jiangsu China

**Keywords:** *CIC*, LGG, mutated genes, prognosis, tumour immune microenvironment

## Abstract

Low‐grade glioma (LGG) is a heterogeneous tumour with the median survival rate less than 10 years. Therefore, it is urgent to develop efficient immunotherapy strategies of LGG. In this study, we analysed mutation profiles based on the data of 510 LGG patients from the Cancer Genome Atlas (TCGA) database and investigated the prognostic value of mutated genes and evaluate their immune infiltration. Tumor Immune Dysfunction and Exclusion (TIDE) algorithm was used to indicate the characteristics of gliomas that respond to immune checkpoint blockade (ICB) therapy. Univariate and multivariate cox regression analysis was performed to identify indicators to construct the nomogram model. 485 (95.47%) of 508 LGG samples showed gene mutation, and 9 mutated genes were significantly related to overall survival (OS), among which 6 mutated genes were significantly correlated with OS between mutation and wildtypes. Immune infiltration and immune score analyses revealed that these six mutated genes were significantly associated with tumour immune microenvironment in LGG. The response of LGG with different characteristics to ICB was evaluated by TIDE algorithm. Finally, *CIC* gene was screened through both univariate and multivariate Cox regression analyses, and the nomogram model was established to determine the potential prognostic value of *CIC* in LGG. Our study provides comprehensive analysis of mutated genes in LGG, supporting modulation of mutated genes in the management of LGG.

## 1 INTRODUCTION

Low‐grade glioma (LGG), a heterogeneous tumour constituting up to approximately 20% of intracranial tumours,^1^ is one of the most popular primary intracranial tumours in humans. LGG is classified by the World Health Organization (WHO) as grades I and II according to clinicopathological and histological features.^1^ WHO grade I LGG is benign and discrete tumours that can be cured by surgical removal, and WHO grade II LGG is diffuse, infiltrative and rarely curable.^2^ Most LGG cases occur in the fourth decade of life, and the median survival rate of LGG patients is less than 10 years.^3^ Seizure is the most common initial symptom of LGG, which seriously threatens the life quality of patients.^4^ In general, the current standard therapy of LGG is a combination of maximal safe surgical resection, radiation therapy, chemotherapy and antiepileptic drugs.^5^ In recent years, people have made unprecedented progress in understanding the genomic changes and molecular biology of gliomas, which provides a large‐scale collaborative treatment trial, but the improvement of survival rate is still unsatisfactory because of gliomas highly invasive characteristic and treatment resistance.^6^, ^7^, ^8^, ^9^ Therefore, understanding the molecular mechanisms to find novel therapeutic methods plays a vital role in delaying tumour malignancy transformation and prolonging survival time of patients with LGG.

The tumour microenvironment (TME) represents the circumstance in which tumour cells grow, and it is composed of tumour cells themselves and a few kinds of stromal cells, including the fibroblasts, immune and inflammatory cells, glial cells and other cells surrounding them.^10^ Infiltrating immune cells are an essential part of the tumour microenvironment, and they play a critical role in tumorigenesis and tumour progression.^11^ It has been reported that significant changes in critical components of the immune response can subsequently lead to tumour immune evasion in gliomas.^12^ Currently, immunotherapy for many types of cancers is a promising strategy to revolutionize clinical management and improve survival rate of patients with LGG. Different from conventional therapies, immunotherapy can activate the patient's autoimmune system and enhance the patient's anti‐tumour immune response to kill cancer cells.^13^ Recently, different immunotherapies have been used to treat many malignant cancers, including immune checkpoint blockade (ICB), therapeutic vaccines, cytokine therapy and cellular therapy.^14^, ^15^ Tumour mutational burden (TMB) and microsatellite instability (MSI) are well‐characterized biomarkers predicting of responses to immunotherapies.^16^, ^17^ Previous study had provided new insights into LGG immunotherapies through exploring the relationship between mutants of the hub genes and immune cell infiltration and the relationships between the immune‐related risk score system and the TME in LGG using informatic analysis; the result showed the negative correlation between TMB and OS and the relationship between high TMB and worse immune infiltration in LGG.^18^ In order to gain precision medicine and a better prognosis in immunotherapy, it is thus meaningful to further investigate the correlations between the mutated genes and tumour immunity features, and explore reliable biomarkers of immunotherapy against LGG.

It is thus necessary to perform the feasible bioinformatical analysis for clinical information as strong evidence to identify the core molecules and predict the prognosis of LGG. In this study, we analysed the mutation profiles based on the data of 510 LGG patients from TCGA database and screened top 30 mutated genes with the highest mutation frequency, followed by functional enrichment analysis through Gene Ontology (GO) term and Kyoto Encyclopedia of Genes and Genomes (KEGG) pathway enrichment analysis. We also investigated the effect of the mutated gene expression level on the overall survival (OS) rate of patients with LGG, revealing nine mutated genes expression level significantly correlated with the OS of LGG patients. Subsequently, we investigated the expression level of these mutated genes in gliomas and normal samples and the effect of these mutated genes on the OS in mutation and wildtypes, indicating among which 6 mutated genes were significantly correlated with OS in both mutation and wildtypes. We analysed the relationship between mutated genes and immune infiltration parameters. We also performed Tumor Immune Dysfunction and Exclusion (TIDE) algorithm to indicate the characteristics of gliomas that respond to immune checkpoint blockade (ICB) therapy. *CIC* gene was identified as a potential independent indicator through both univariate and multivariate Cox regression analyses with the inclusion of prognostic value of age, gender and grade. Finally, a predictive nomogram model of LGG was constructed to evaluate the potential prognostic value of *CIC* in LGG. The present study may be helpful to developing targeted drugs and immunosuppressants for the treatment of patients with LGG, and provide a new idea and direction for the clinical research of LGG therapy.

## 2 MATERIALS AND METHODS

### 2.1 Data acquisition

The genetic mutation data, transcriptome data and clinical data of TCGA‐LGG dataset were obtained from the Cancer Genome Atlas (TCGA) database (http://cancergenome.nih.gov/abouttcga). The clinical data of patients are shown in Table S1. Additionally, RNA‐seq data from Chinese Glioma Genome Atlas (CGGA) database (http://www.cgga.org.cn/) and REMBRANDT dataset from GlioVis (http://gliovis.bioinfo.cnio.es/) were downloaded as validation cohorts. All data retrieved from TCGA and GTEx database were corrected and normalized using the ‘normalize between array’ function of the ‘limma’ R package.

### 2.2 Mutation landscape in the LGG cohort

To comprehensively investigate the somatic mutations of the patients with LGG in the TCGA database, mutation data were downloaded and visualized using the ‘maftools’ R package through R software (version 4.0.3).

### 2.3 Functional enrichment analysis of mutated genes in LGG

To obtain a comprehensive set of functional annotation for top 30 mutated genes in LGG, R package ‘clusterProfiler’ in R software (version 4.0.3)^19^ was employed to investigate the GO and KEGG enrichment analysis of top mutated genes in LGG in this study with FDR < 0.05 as the cut‐off criteria.

### 2.4 Identification of survival‐related mutated genes

The TCGA‐LGG dataset was used to identify mutated genes associated with OS. The Kaplan‐Meier survival analysis with log‐rank test was used to compare the survival difference between high and low expression of mutated genes in LGG. Meanwhile, we also investigated the OS difference between mutant and wildtype group of mutated genes in LGG. In addition, GSCA database (http://bioinfo.life.hust.edu.cn/GSCA/ #/) was performed to determine the SNV (Single Nucleotide Variation) and CNV (Copy Number Variation) of mutated genes, and their correlation with survival. In addition, we further explored the survival difference between mutated gene set SNV and wildtype. For Kaplan‐Meier curves, *p*‐values and hazard ratio (HR) with 95% confidence interval (CI) were generated by log‐rank tests and univariate Cox proportional hazards regression. Expression distribution difference of survival‐related genes between LGG samples and normal tissues was performed by the Kruskal‐Wallis test. Meanwhile, we also investigated the expression difference of survival‐related genes on genders and ages in patients with LGG.

### 2.5 Identification of the correlation between survival‐related mutated genes and immune infiltrates

To observe the differences of immune cells in LGG samples and normal tissues, GSCA database was first used to explore the correlation between immune infiltrates and expression, SNV and methylation of survival‐related mutated genes. Then, we further investigated the immune cell score in mutant and wildtype of survival‐related mutated genes using R package ‘immunedeconv’ through TIMER algorithms, in R software (version 4.0.3).^20^ *SIGLEC15*, *TIGIT*, *CD274*, *HAVCR2*, *PDCD1*, *CTLA4*, *LAG3* and *PDCD1LG2* were selected to be immune checkpoint‐related genes, and their expression values in mutant and wildtype of survival‐related mutated genes in LGG were extracted. The expression correlation between the expression of survival‐related mutated genes and that of immune checkpoints‐related genes in LGG was depicted with Spearman's correlation analysis.

### 2.6 Correlation analysis between the expression of survival‐related mutated genes and TMB/MSI

We analysed the correlation between the expression of survival‐related mutated genes and TMB/MSI. We used Spearman's correlation analysis to describe the correlation, and a *p*‐value of less than 0.05 was considered statistically significant.

### 2.7 Immune checkpoint blockade response prediction using TIDE algorithm

Tumor Immune Dysfunction and Exclusion algorithm^21^ uses a set of gene expression markers to evaluate the mechanism of tumour immune escape. Here, potential immune checkpoint blockade (ICB) response was predicted with TIDE algorithm based on expression profile in LGG. The high TIDE score indicates the bad curative effect of ICB and short survival time after ICB treatment in patients.

### 2.8 Identification of independent prognostic parameters and construction of predictive Nomogram for LGG

To further identify independent prognostic factors and to validate the independent prognostic value of mutated genes, we first used univariate Cox and log‐rank tests to evaluate the prognostic significance of OS and progress‐free survival (PFS). In addition, multivariate Cox regression analyses were further performed in the TCGA‐LGG dataset on the prognostic mutated genes and clinicopathological parameters including age, sex and tumour grade. Based on univariate and multivariate Cox regression analyses, predictive Nomograms of OS and PFS for LGG were constructed through ‘rms’ R package.

### 2.9 Statistical analysis

All the above analysis methods and R packages were implemented by R foundation for statistical computing (2020) version 4.0.3 and ggplot2 (v3.3.2). *P* value < 0.05 was considered statistically significant.

## 3 RESULTS

### 3.1 Mutation landscape of somatic mutations in LGG

**FIGURE 1:**
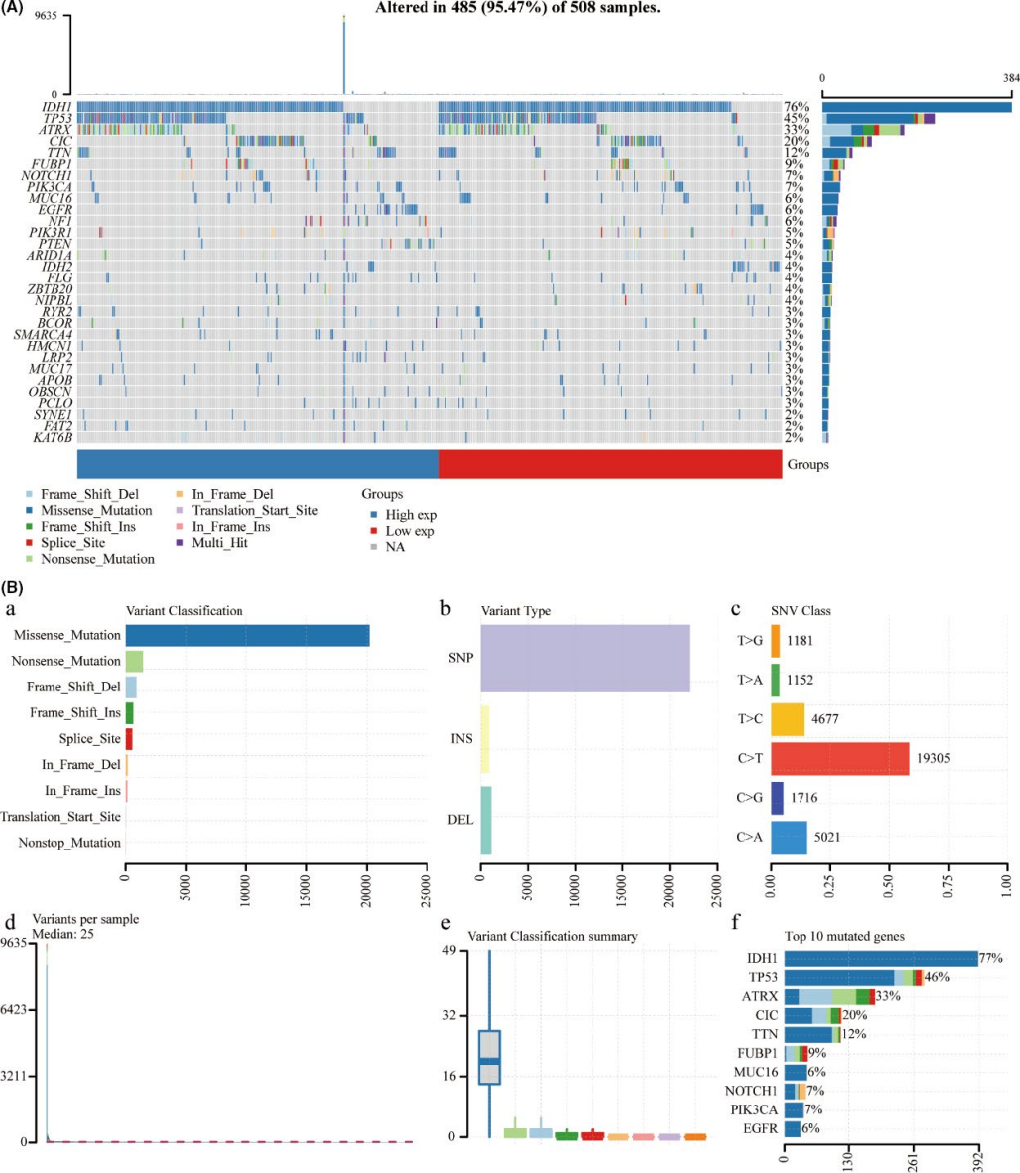
Mutation landscape of the low‐grade glioma (LGG) cohort. Waterfall plot showing the mutation patterns of top 30 mutated genes ordered by the mutation frequency, with different colours with specific annotations at the bottom representing the various mutation types (A). Cohort summary plot displays classification of mutation types according to different categories and tumour mutation burden in samples (B)

We downloaded mutation data from TCGA database and used the ‘maftools’ package in R software to visualize the data so as to identify the somatic mutations of the patients with LGG. Horizontal histogram showed the top 30 mutated genes with the highest mutation frequency in patients with LGG (*n* = 508; Figure 1A). The most common type of mutation in LGG patients was missense mutations (Figure 1Ba). Single nucleotide polymorphism (SNP) was the predominant mutation variant type (Figure 1Bb), and C>T occupied an absolute position compared with other SNV classes (Figure 1Bc). The median of the mutation variants per sample was 25 as shown in Figure 1Bd, and the box diagram of each colour represents a kind of mutation as shown in Figure 1Be. In addition, Figure 1Bf showed the top 10 mutated genes, including *IDH1* (76%), *TP53* (45%), *ATRX* (33%), *CIC* (20%), *TTN* (12%), *FUBP1* (9%), *MUC16* (7%), *NOTCH1* (7%), *PIK3A* (6%) and *EGFR* (6%). In the following analysis, we focused on these top 30 mutated genes with the highest mutation frequency.

### 3.2 Functional enrichment analysis of top 30 mutated genes

Three categories of GO functional enrichment analyses (biological process (BP), cellular component (CC) and molecular function (MF)) and KEGG pathway enrichment analysis were performed to further analyse the function of the top 30 mutated genes with the highest mutation frequency shown in the Figure 1A. The enriched GO terms for these 30 mutated genes mainly included the striated muscle tissue development, muscle tissue development, cardiac muscle tissue development, heart morphogenesis and phosphatidylinositol 3‐kinase signalling items in the BP category (Figure S1A), apical plasma membrane, apical part of cell, sarcomere, contractile fibre part and myofibril items in the CC category (Fig. S1B), and chromatin binding, calmodulin binding and protein self‐association items in the MF category (Figure S1C). As shown in Figure S1D and S1E, the KEGG pathway analysis indicated that central carbon metabolism in cancer, hepatocellular carcinoma, endometrial cancer, melanoma and breast cancer were the mainly enriched pathways in the top 30 mutated genes. Figure S1F showed the association between the main mutated genes (*PIK3R1*, *PIK3CA*, *TP53*, *EGFR*, *PTEN*, *NOTCH1*, *SMARCA4*, *NF1*, *IDH1* and *ARID1A*) and the top 20 KEGG terms. Figure S2 showed the enrichment network describing the association among the different GO term such as BP (Figure S2A), MF (Figure S2B) and CC (Figure S2C) and the association among the enriched KEGG pathways (Figure S2D). Moreover, dot‐line network plots display the relationship of mutated genes with the top 10 enriched biological processes (Figure S2E) and the top 10 enriched KEGG pathways (Figure S2F).

### 3.3 Prognostic value of the mutated genes in LGG

**FIGURE 2:**
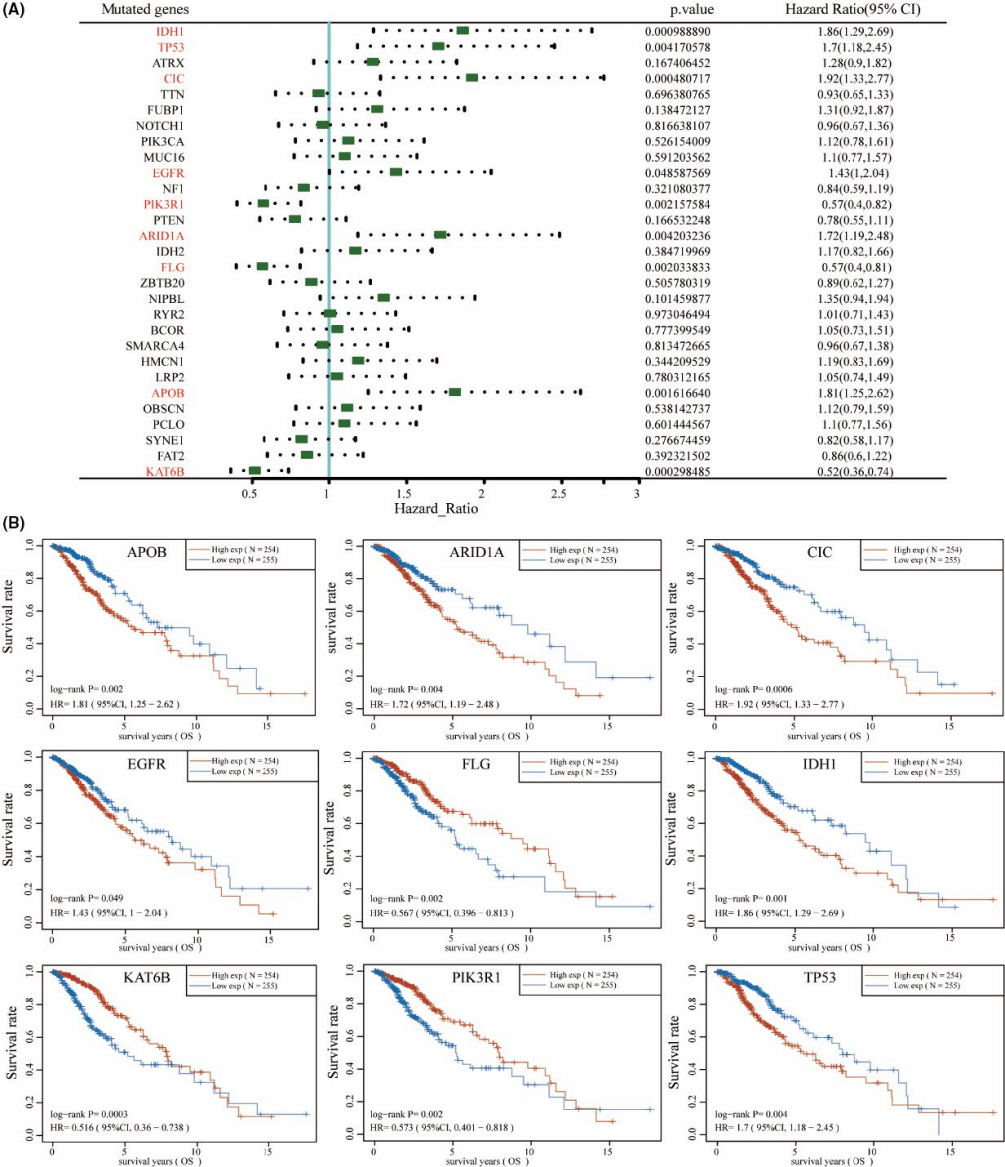
Survival analysis of the mutated genes. Survival analysis of top 30 mutated genes was performed by the log‐rank tests and univariate Cox proportional hazards regression (A), and the nine mutated genes (*APOB*, *ARID1A*, *CIC*, *EGFR*, *FLG*, *IDH1*, *KAT6B*, *PIK3R1* and *TP53*) are related to OS (overall survival) in LGG (B)

Next, we downloaded the prognostic information of the patients with LGG from TCGA database to analyse the prognostic value of the top 30 mutated genes by the log‐rank tests and univariate Cox proportional hazards regression, and the results showed that the expression level of 9 mutated genes was significantly correlated with survival prognosis, including *IDH1*, *TP53*, *CIC*, *EGFR*, *PIK3R1*, *ARID1A*, *FLG*, *APOB* and *KAT6B* (Figure 2A). Then, we performed Kaplan‐Meier analysis to analyse the prognostic value of these 9 mutated genes, and the results showed that high expression of *APOB*, *ARIDIA*, *CIC*, *EGFR*, *IDH1* and *TP53* was associated with worse OS (*p* < 0.05 for EGFR, and *p* < 0.01 for the other genes) and high expression of *FLG*, *KAT6B* and *PIK3R1* was significantly related with a better prognosis in patients with LGG (*p* < 0.01 for all the three genes; Figure 2B). We also downloaded the prognostic information of the patients with LGG from REMBRANDT database and CGGA database to analyse the prognostic value of the screened genes by Kaplan‐Meier analysis. As shown in the Figure S3A, high expression of *ARIDIA*, *CIC*, *EGFR*, *IDH1* and *TP53* was associated with worse OS (*p* < 0.05 for *ARIDIA* and *CIC*, and *p* < 0.01 for the other genes) and high expression of *APOB*, *FLG*, *KAT6B* and *PIK3R1* was significantly related with a better prognosis in patients with LGG (*p* < 0.05 for *APOB* and *FLG*, and *p* < 0.01 for the other genes) in the REMBRANDT database. As shown in the Figure S3B, high expression of *APOB*, *ARIDIA*, *CIC*, *EGFR*, *IDH1* and *TP53* was associated with worse OS (*p* < 0.05 for *TP53*, and *p* < 0.01 for the other genes) and high expression of *FLG*, *KAT6B* and *PIK3R1* was significantly related with a better prognosis in patients with LGG (*p* < 0.05 for *KAT6B* and *FLG*, and *p* < 0.01 for *PIK3R1*) in the CGGA database.

### 3.4 The expression of the mutated genes in LGG and GBM

Subsequently, we assessed the relative expression of the nine mutated genes in the LGG, GBM and normal samples. Analysis of expression profiles showed that the expression of *ARIDIA*, *CIC*, *EGFR*, *IDH1*, *KAT6B*, *PIK3R1* and *TP53* was significantly higher in LGG and GBM patients compared with normal samples (*p* < 0.001 for all the genes) and *FLG* expression in LGG was significantly higher than that of normal samples (*p* < 0.001; Figure S4A). Only the expression of *APOB* had no significant difference in the LGG, GBM and normal samples. We also found the expression level of *EGFR* and *CIC* in the male is higher than that in the female (Figure S4B). In addition, only *EGFR*, *FLG* and *KAT6B* expression level was significantly altered in LGG patients of different ages, compared to other mutated genes (Figure S4C).

### 3.5 Survival analysis of the nine mutated genes in mutant groups and wildtypes

**FIGURE 3:**
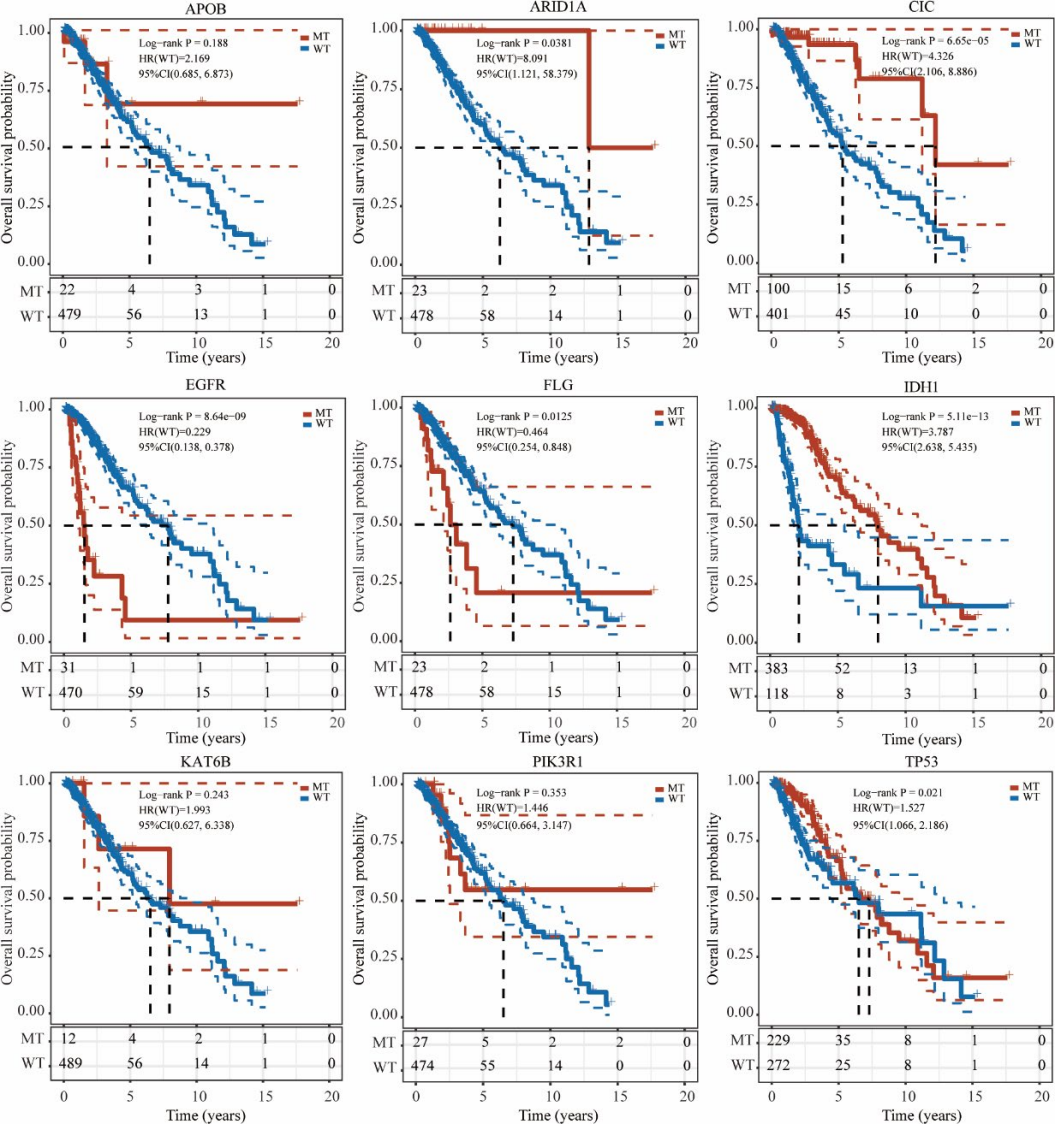
Prognostic analysis of nine mutated genes in mutant and wild type. Mutations in *ARID1A*, *CIC*, *EGFR*, *FLG*, *IDH1* and *TP53* have a prognostic impact in patients with Low‐grade glioma (LGG). MT, mutant type; WT, wildtype

To further explore the prognostic value of the screened mutated genes, we also analysed the OS of the corresponding mutant and wildtypes. In addition, we found that *ARID1A* mutant group (*p* < 0.05, HR(WT) = 8.091, 95%CI (1.121, 58.379)), *CIC* mutant group (*p* < 0.001, HR(WT) = 4.326, 95%CI (2.106, 8.886)) and *IDH1* mutant group (*p* < 0.001, HR(WT) = 3.787, 95%CI (2.638, 5.435)) showed significantly better OS than wildtype. In addition, the OS of *EGFR* mutant group (*p* < 0.001, HR(WT) = 0.229, 95%CI (0.138, 0.378)), *FLG* mutant group (*p* < 0.05, HR(WT) = 0.464, 95%CI (0.254, 0.848)) and *TP53* mutant group (*p* < 0.05, HR(WT) = 1.527, 95%CI (1.066, 2.186)) was significantly lower than wildtype, whereas *APOB*, *KAT6B* and *PIK3R1* had no significant difference for OS in mutant and wildtypes (Figure 3).

### 3.6 Copy number variation and SNV analyses of six mutated genes in LGG

**FIGURE 4:**
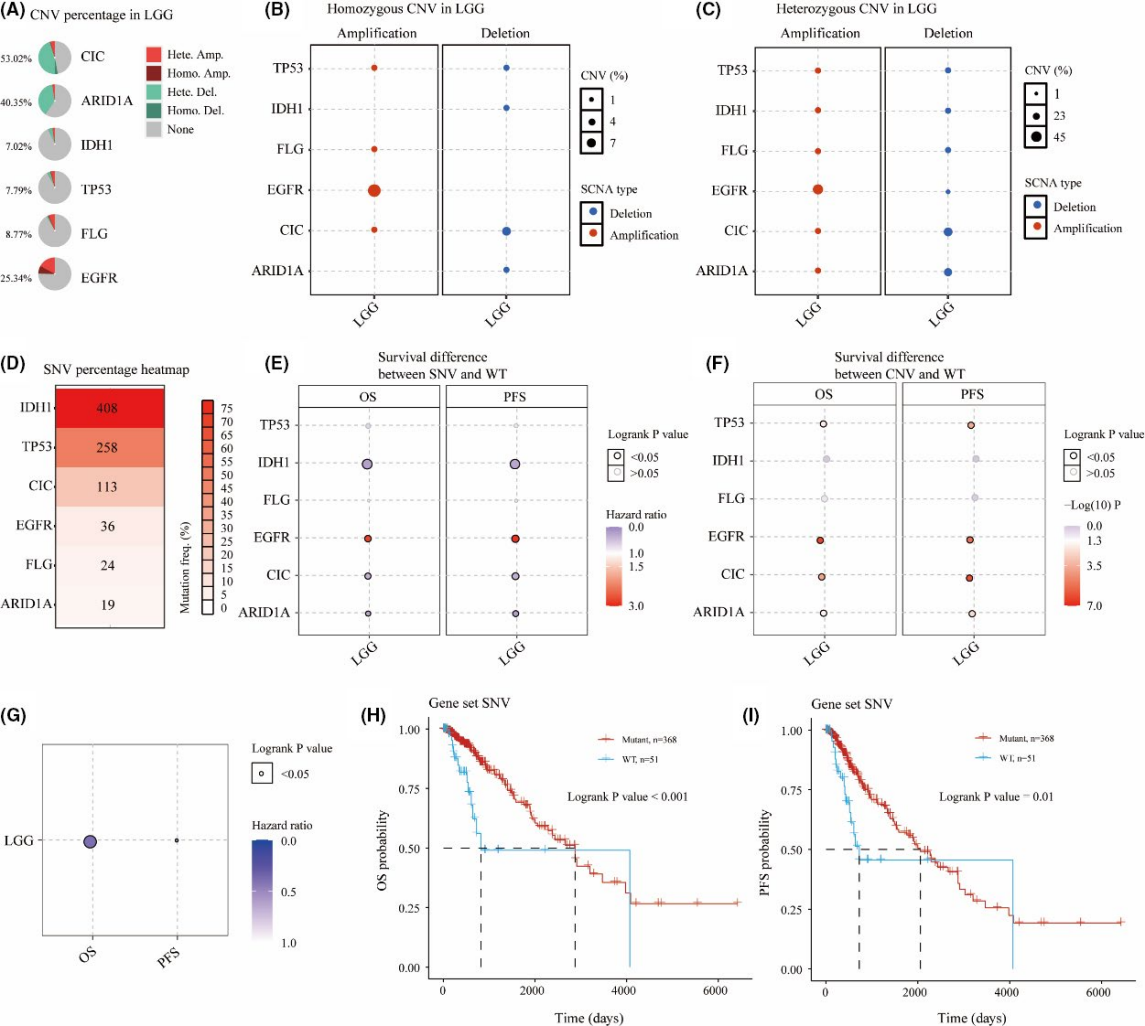
Copy number variation (CNV) and single nucleotide variation (SNV) analyses of six mutated genes in Low‐grade glioma (LGG). CNV percentage of six mutated genes in LGG (A). Homozygous and heterozygous CNV of six mutated genes in LGG (B, C). SNV percentage of six mutated genes in LGG (D). OS and PFS difference between SNV/CNV and WT in LGG (E, F). Survival difference between Gene set SNV and WT (G). OS and PFS probability of gene set SNV in LGG (H, I)

Next, we performed genetic variants analysis of the screened six mutated genes in LGG using GSCA database, including CNV and SNV. We analysed the CNV percentage of six survival‐related mutated genes in LGG, demonstrating that *CIC* gene had the highest CNV percentage (53.02%), followed by *ARID1A* (40.35%), *EGFR* (25.34%), *FLG* (8.77%), *TP53* (7.79%) and *IDH1* (7.02%; Figure 4A). From the perspective of heterozygous CNV in LGG, heterozygous amplification is the main alteration in *TP53*, *FLG* and *EGFR* and heterozygous deletion is the main alteration in *CIC*, *ARID1A* and *IDH1* (Figure 4B). From the perspective of homozygous CNV in LGG, homozygous amplification is the main alteration in *EGFR* and homozygous deletion is the main alteration in *CIC* (Figure 4C). As shown in Figure 4D, we also analysed the SNV percentage of six mutated genes in LGG, demonstrating that *IDH1* gene had the highest SNV, followed by *TP53*, *CIC*, *EGFR*, *FLG* and *ARID1A*.

Then, we compared the OS between SNV group and wildtype, and CNV group and wildtype. As shown in Figure 4E, *EGFR*‐SNV group, *IDH1*‐SNV group, *CIC*‐SNV group and *ARID1A*‐SNV group were significantly different from wildtype in OS and PFS, among which *EGFR*‐SNV group had the highest risks in OS and PFS. As shown in Figure 4F, *TP53*‐CNV group, *EGFR*‐CNV group, *CIC*‐CNV group and *ARID1A*‐CNV group were significantly different from wildtype in OS and PFS. We then integrated the sample set of mutated genes with SNV as a whole gene set and compared their OS and PFS with the wildtype. We found that the OS and PFS of mutated gene set with SNV were obviously different compared with the wildtype (Figure 4G). Better OS and PFS were observed in the gene set with SNV than in the wildtype during the follow‐up period of 2000 days (Figure 4H‐I).

### 3.7 The immune cell infiltration of the six mutated genes in LGG

**FIGURE 5:**
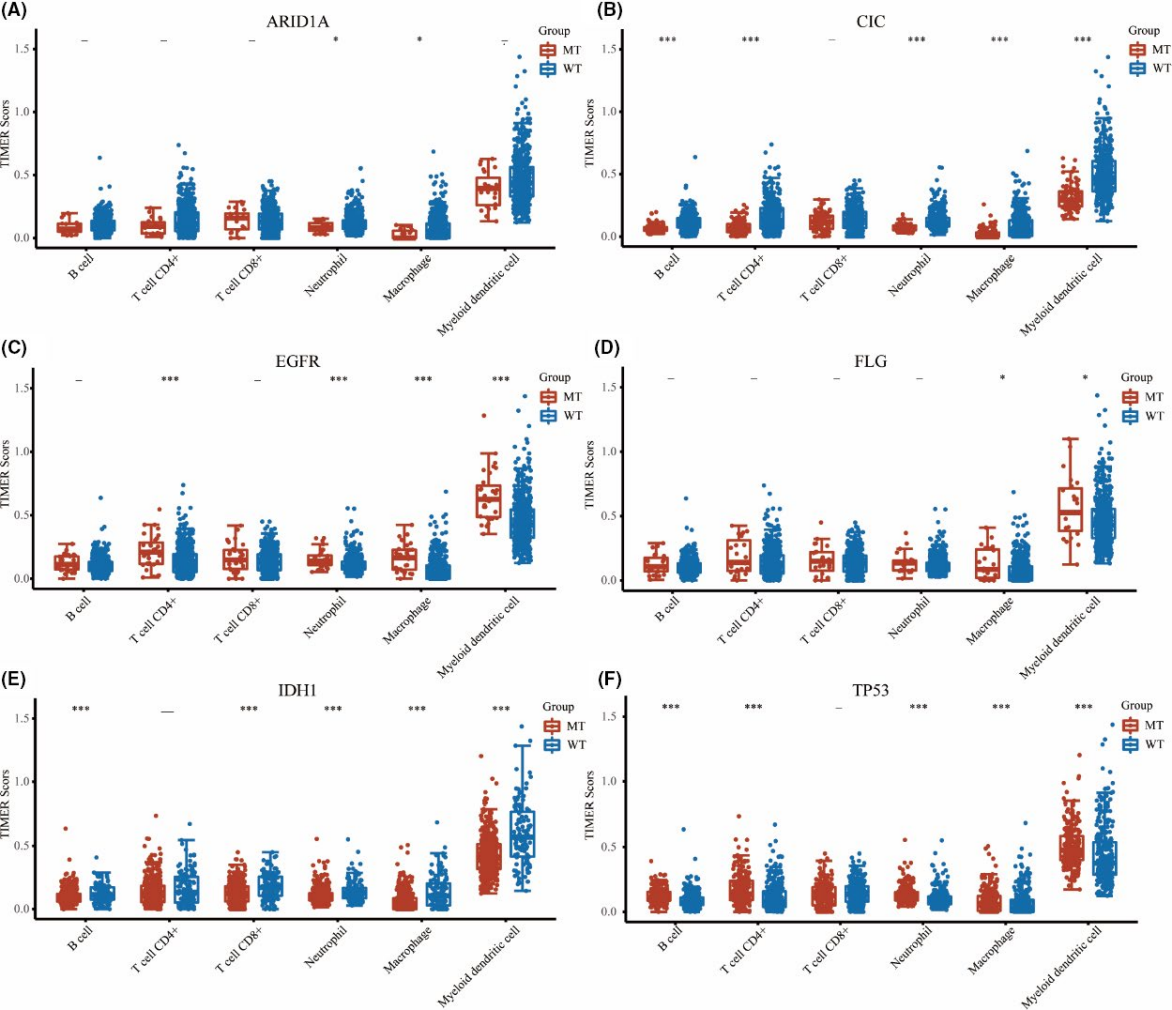
Immune cell score. TIMER score of immune cells in mutant and wildtypes of six hub mutated genes including *ARID1A* (A), *CIC* (B), *EGFR* (C), *FLG* (D), *IDH1* (E) and *TP53* (F). MT, mutant type; WT, wildtype. The significance of two groups of samples passed the Kruskal‐Wallis test, ‐, no significance; *p* > 0.05; *, *p* < 0.05; ***, *p* < 0.001

To further explore the differences of immune cell infiltration in LGG samples and normal tissues, GSCA database was used to analyse the correlation between immune cell infiltration and expression, methylation and SNV of six survival‐related mutated genes. As shown in Figure S5A, the expression level of the most mutated genes was significantly positively correlated with immune infiltration of most immune cells, such as B cells, macrophage, monocyte, DC cells, neutrophil and central memory cells, and significantly negatively correlated with immune infiltration of a few immune cells, such as CD4 T cells, MAIT cells, NK cells, CD8 T cells and CD8 naive cells. Figure S5B showed the association between methylation of survival‐related mutated genes and immune cell infiltration. It was obvious that most methylation of survival‐related mutated genes was significantly negatively correlated with immune infiltration of a lot of immune cells, such as cytotoxic cells, NK cells, Tfh cells, Th2 cells, Tr1 cells, DC cells, macrophage, effector memory cells, monocyte and Th1 cells. Figure S5C showed difference of immune infiltrates between SNV and wildtype of survival‐related mutated genes, and the result showed that SNV of *EGFR*, *TP53*, *CIC* and *IDH1* in LGG resulted in the slight change of immune infiltration. Moreover, immune cell score in mutant and wildtype of survival‐related mutated genes was investigated using R package ‘immunedeconv’ through TIMER algorithms, in R software. The results showed that the immune score of neutrophil cells and macrophage cells in *ARID1A* mutant group was significantly lower than that in wildtype (Figure 5A). The TIMER scores for B cell, CD4+ T cell, neutrophil cell, macrophage cell and myeloid dendritic cell of the wildtype were significantly higher than those of the *CIC* mutant group (Figure 5B). The following immune infiltrating cells were considerably higher in *EGFR* mutant group compared to wildtype, that is CD4+ T cell, neutrophil cell, macrophage cell and myeloid dendritic cell (Figure 5C). In addition in *FLG* mutant group, only the immune scores of macrophage cell and myeloid dendritic cell were significantly higher than those in wildtype (Figure 5D). A higher number of immune‐associated cells such as B cell, CD8+ T cell, neutrophil cell, macrophage cell and myeloid dendritic cell were observed in wildtype than in *IDH1* mutant group (Figure 5E), while B cell, CD4+ T cell, neutrophil cell, macrophage cell and myeloid dendritic cell responded more pronouncedly to *TP53* mutation (Figure 5F).

### 3.8 Immune checkpoints analysis in LGG

We next analysed the correlation between the expression level of these six hub mutated genes and the expression of immune checkpoints‐related genes in LGG. As shown in Figure S6A, the expression levels of *CIC* and *ARID1A* were significantly positively correlated with those of *CD274*, *CTLA4*, *HAVCR2*, *LAG3*, *PDCD1* and *PDCD1LG2*, whereas negatively correlated with that of *TIGIT*. A significantly positive correlation was found between *EGFR* and the following immune checkpoints, that is *CD274* and *PDCD1LG2*, and between *FLG* and two immune checkpoints proteins *CD274* and *TIGIT*, and a significantly negative correlation was found between *FLG* and *LAG3* in LGG. In addition, IDH1 expression was in direct proportion to those of genes *CTLA4*, *HAVCR2*, *LAG3*, *PDCD1*, *PDCD1LG2* and *TIGIT*. The expression level of *TP53* was significantly positively correlated with those of *LAG3*, *PDCD1* and *SIGLEC15*.

Next, we further analysed the correlation between the expression levels of the six mutated genes and those of immune checkpoints genes in both the mutant and wildtype. As shown in Figure S6Ba, the expression level of *PDCD1LG2* in *ARID1A* mutant group was significantly inhibited compared with the wildtype. The wildtype showed higher expression of *CD274*, *CTLA4*, *HAVCR2*, *PDCD1* and *PDCD1LG2* and lower expression of *TIGIT* than the *CIC* mutant group (Figure S6Bb) and showed higher expression of all of the above genes than *IDH1* mutant group (Figure S6Be). Except for *LAG3* and *TIGIT*, the expression of *CD274*, *CTLA4*, *HAVCR2*, *PDCD1*, *PDCD1LG2* and *SIGLEC15* was higher in mild group than in *EGFR* mutant group (Figure S6Bc). The expression levels of *CTLA4*, *HAVCR2*, *PDCD1* and *PDCD1LG2* in the *FLG* mutant group were significantly higher than the wildtype (*p* < 0.01 for *CTLA4* and *p* < 0.05 for the other genes; Figure S6Bd). TP53 mutation in LGG resulted in higher expression of six immune checkpoint‐related genes including *CD274*, *HAVCR2*, *LAG3*, *PDCDILG2*, *TIGIT* and *SIGLEC15* (Figure S6Bf).

### 3.9 Correlation of six mutated gene expression levels with TMB and MSI scores in LGG

**FIGURE 6:**
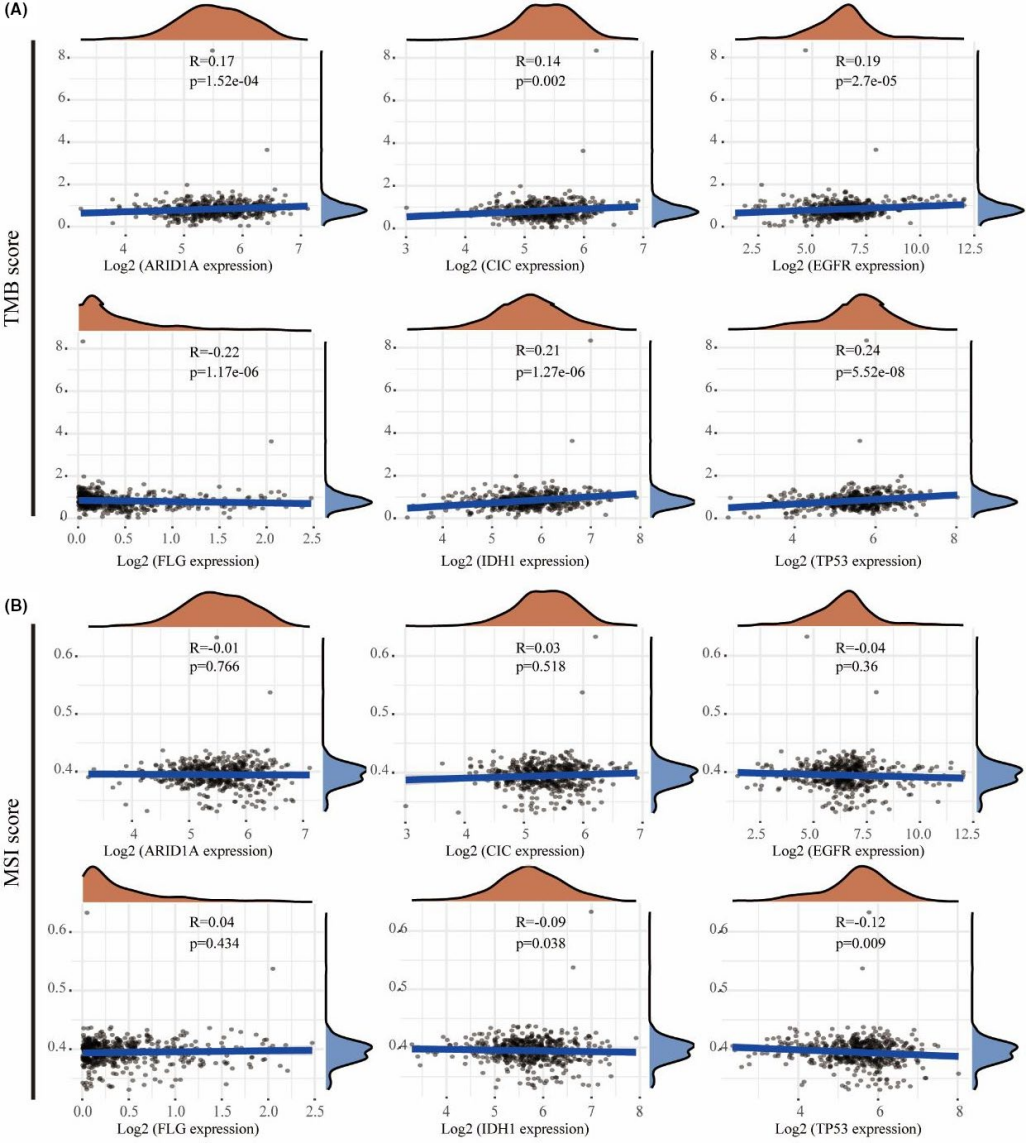
Correlation of six hub mutated gene expression levels with Tumour mutational burden (TMB) (A) and MSI (B) score in low‐grade glioma (LGG)

Tumour mutational burden and MSI have been the main prognostic biomarkers over the last few years, and their prognostic value in LGG remains uncertain. Using multi‐omics data from TCGA, we systematically analysed the correlations between TMB/MSI and the expression level of six mutated genes in LGG to identify the influence of these six mutated genes in the development of LGG. As shown in Figure 6A, six genes were significantly positively correlated with the TMB score in LGG, whereas only *IDH1* and *TP53* were significantly negatively correlated with MSI, and there was no significant relationship between other mutated genes and MSI (Figure 6B).

### 3.10 Tumour immune dysfunction and exclusion (TIDE) signature predicts ICB response

Next, TIDE score was employed to predict the response to ICB therapy in different samples to the predicted immune checkpoint inhibitors. We compared the TIDE scores between the high‐expression groups and low‐expression groups of six survival‐related mutated genes in LGG (Figure S7A‐F), and between wild types and mutant groups of six survival‐related mutated genes in LGG (Figure S7G‐L) to predict potential immunotherapeutic responses. High expression of *ARID1A* had lower TIDE score compared with low expression of *ARID1A* (*p* < 0.01; Figure S7A). The TIDE score was significantly lower in samples with low expression of *IDH1* and *TP53* than those with high‐expression of *IDH1* and *TP53* (*p* < 0.0001; Figure S7E,F). The TIDE scores in *CIC*‐wildtype and *EGFR*‐wildtype groups were significantly lower than in *CIC* and EGFR‐mutant groups (*p* < 0.001 for *CIC* and *p* < 0.05 for *EGFR*; Figure S7H,I). The TIDE scores in *IDH1* and *TP53* mutant groups were significantly lower than that in wildtype *IDH1* and *TP53* groups (*p* < 0.0001; Figure S7K,L).

### 3.11 Prognostic value of the hub mutated genes in LGG

**FIGURE 7:**
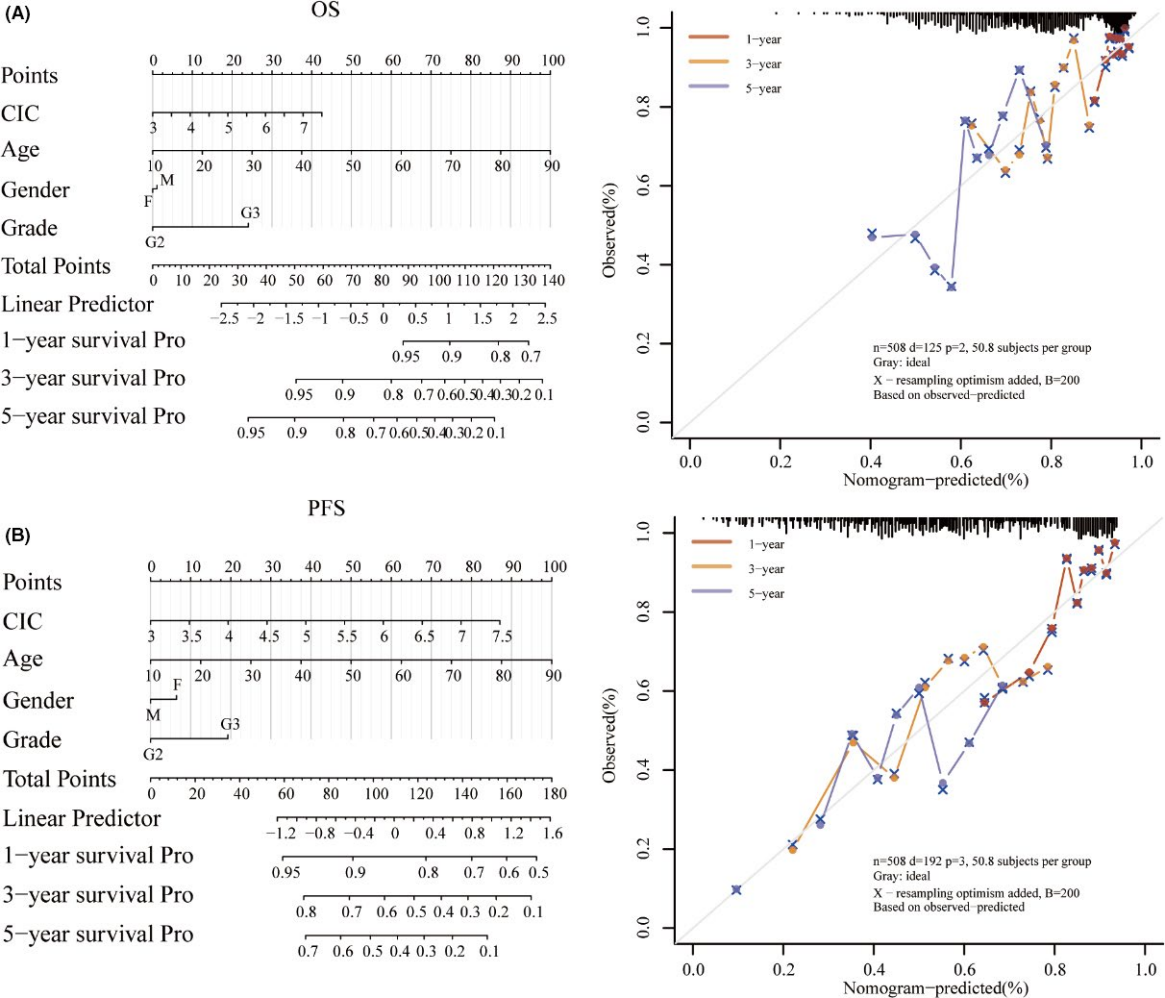
Construction of the nomogram based on TCGA‐LGG dataset. Nomogram to predict the 1‐, 2‐ and 3‐year OS (A) and progression‐free survival (B) of patients with LGG. A dashed diagonal line represents the ideal nomogram, and the orange, yellow and blue lines represent the 1‐, 3‐ and 5‐year observed nomograms, respectively

According to results above, we selected the five mutated genes (*IDH1*, *TP53*, *FLG*, *CIC* and *EGFR*) to confirm their prognostic value in LGG. Both univariate and multivariate Cox regression analyses were conducted with the inclusion of prognostic value of age, gender and grade, which showed that only *CIC* was significantly associated with both OS and PFS of LGG patients (Table. S2), suggesting *CIC* was independent prognostic factors in LGG. Therefore, with the inclusion of clinical relevance and prognostic value of *CIC* expression signature, a prognostic nomogram was constructed as a clinically dependable predictive method for predicting the survival probability of 1‐, 3‐ and 5‐year OS and PFS of patients with LGG. The nomogram provides a graphical representation of *CIC* expression, and the OS (Figure 7A) and PFS (Figure 7B) of a single patient can be calculated by the points associated with *CIC* expression level, age, gender and WHO grade.

## 4 DISCUSSION

Gliomas were classified in four grades according to clinicopathological and histological features by the WHO.^22^ WHO grades I and II are classified as LGG, while the other two grades III and IV are classified as high‐grade glioma (HGG).^23^ LGG was characterized by slow progress and malignant transformation potential.^5^ Virtually, all LGG finally will be malignantly transformed to HGG.^24^ Seizure is the initial symptom of patients with LGG that seriously threatens human life quality.^4^ Thus, identifying novel LGG‐related molecular mechanisms and finding effective molecular targeted therapies play an important role in improving the survival quality of patients with LGG.

In our study, the evidence from the analysis of the landscape of mutation profiles showed that 95.47% of patients with LGG contain diverse types of mutations. We found that *IDH1* had the highest mutation frequency in patients with LGG (76%), followed by *TP53*, *ATRX*, *CIC*, *TTN*, *FUBP1*, *NOTCH1*, *PIK3CA*, *MUC16* and *EGFR*. It was reported that high percentages of *IDH1* and *IDH2* mutations (70%–80%) had been found in a high percentage of LGG and less in high‐grade gliomas,^25^, ^26^ and *TP53* mutations are observed in about 30% of gliomas, mainly LGG,^27^ which was similar to the results in the present study. The discovery of *IDH1* and *IDH2* mutations led to a biomarker defined classification of gliomas based on prognosis and treatment response.^28^ Previous researches have indicated that the close relationship between *IDH1* mutation and high survival rate also suggests that *IDH1* may be a new epigenetic target for glioma therapy.^29^, ^30^ Mutation in *ATRX* also had been reported, commonly in LGG.^31^ In addition, we also found the mutations of these mutated genes in the patients with LGG were mainly missense mutations. A high proportion of missense mutations may change genetic information though affecting the structure and function of proteins,^32^, ^33^ suggesting possible roles in the pathogenesis of LGG. SNP was the main mutation variant type, and C>T was the main DNA base substitution compared with other SNV classes in the patients with LGG. Then, we performed KEGG pathway enrichment analysis, showing that top 30 screened genes in LGG were enriched in items including central carbon metabolism in cancer, hepatocellular carcinoma, endometrial cancer, melanoma and breast cancer. Next, we performed Kaplan‐Meier survival analysis of the top 30 screened genes, and we found the expression of nine genes was associated with survival rate in patients with LGG. The higher expression of *ARID1A*, *CIC*, *EGFR*, *IDH1* and *TP53* resulted in a lower survival rate of the patients with LGG. The higher expression of the other 3 genes *FLG*, *KAT6B* and *PIK3R1* was significantly associated with a better survival rate in LGG. These analyses suggested *ARID1A*, *CIC*, *EGFR*, *IDH1* and *TP53* may act as tumour promoters in LGG, whereas *FLG*, *KAT6B* and *PIK3R1* may act as tumour suppressors in LGG.

We further compared the expression of these nine genes in normal brain tissues and glioma samples. The expression of *ARID1A*, *CIC*, *EGFR*, *IDH1* and *TP53* in LGG was significantly higher than normal samples, and so are these genes *KAT6B* and *PIK3R1*, indicating that the oncogenic effect of these mutated genes is greater than their tumour inhibition effect. To further study the prognostic role of these nine genes in LGG, we compared the OS difference between mutant group and wildtype, and the results indicated that six mutated genes (*ARID1A*, *FLG*, *IDH1*, *EGFR*, *CIC* and *TP53*) were significantly related to OS in mutation group compared with wildtype. The OS of *ARID1A* mutant group, *CIC* mutant group, *IDH1* mutant group and *TP53* mutant group were significantly higher than that of the wildtype, which is sharply contrasted to the *EGFR* mutant group and *FLG* mutant group, suggesting that different gene mutations may have different effects on the progression of LGG and some mutations are even beneficial to the OS of patients with LGG. Next, we determined the SNV and CNV of mutated genes and explored their correlation with survival using GSCA database. Intriguingly, we found the gene set with SNV had better OS and PFS within 2000 days of follow‐up compared with the wildtype, suggesting that the role of six mutated genes with SNV is not single, but maybe complementary or even beneficial in LGG. It is worth considering whether the relationship between SNV and CNV can be used as an indicator to predict the survival rate of patients with LGG, and to determine the drug development. However, further validation is still required in following studies and clinical trials in patient cohorts.

The TME predominantly consists of infiltrating immune cells and stromal cells, which play an essential role in tumour growth, progression, prognosis and therapeutic approaches.^34^ It is important to explore the interaction between tumour and immunity through the characterization of the immune infiltration landscape.^35^, ^36^ Immune cell infiltration analysis demonstrated that these six genes caused a lot of changes in immune infiltrating cells in LGG. However, methylation of six mutated genes did not cause more drastic changes in the immune microenvironment, thus weakening the infiltration of immune cells. These six genes with SNV induced more slight changes in the infiltration of immune cells. Immune score analysis revealed that six mutated genes were significantly related to the immune microenvironment and correlated with most immune cell infiltration, especially the macrophage cells in LGG, indicating that these mutated genes may contribute to alter immune status and the heterogeneity of immune infiltration was crucial for LGG progression. In addition, we also found these six mutated genes were significantly correlated with the immune score and immune checkpoints. Immune checkpoints are regulatory molecules that play an inhibitory role in the immune system,^37^ and they are essential for maintaining self‐tolerance, preventing autoimmune response, and minimizing tissue damage by controlling the time and intensity of immune response.^38^, ^39^ The function of immune cells can be inhibited by the expression of immune checkpoint molecules on them, which makes the body unable to produce effective anti‐tumour immune response, thus forming immune escape of tumours.^40^ We found that the immune scores and immune checkpoint expression in *CIC*, *IDH1* and *ARID1A* mutant group were obviously lower than that in wildtype, suggesting that the body can better recognize the mutated antigen cells and promote the immune function of T cells to better remove the antigen and inhibit tumour development. The OS values in the *CIC*, *IDH1* and *ARID1A* mutant groups were significantly better than those in the wildtype. Meanwhile, the immune scores and the expression immune checkpoint molecules in *EGFR* and *TP53* mutant groups were obviously higher than those in wildtype, suggesting that *EGFR* and *TP53* mutation may lead to the secretion of certain substances to activate the immune checkpoints, thus inhibiting the immune function of T cells, escaping immune monitoring and promoting tumour progression.

Recently, different immunotherapies have been used to treat many malignant cancers, including ICB, therapeutic vaccines, cytokine therapy and cellular therapy.^14^, ^15^ TMB is another indicator of better response to ICB therapy.^16^, ^17^ TMB, which refers to the total number of somatic protein‐coding base substitution, insertion and deletion mutation, is an important predictive biomarker of immune checkpoint inhibitors in some types of cancers.^41^ Increasing somatic mutations can cause the expression accumulation of neoantigens and tumorigenesis, which results in activation of CD8+ cytotoxic T cells (CTLs) to elicit an anti‐tumour effect of T cell–dependent immune responses.^42^ TMB and MSI have been considered new biomarkers of immunotherapy response and potential predictors of response to immune checkpoint inhibitors (ICIs).^43^, ^44^ The TMB score analysis revealed that the expression level of six survival‐related mutated genes was significantly positively correlated with TMB in LGG. The MSI score analysis revealed that only *TP53* and *IDH1* are conspicuously correlated with MSI in LGG. High TMB scores are considered to result in more neoantigens appearing on the surface of tumour cells, thus increasing immunogenicity and making tumour more sensitive to the treatment using immune checkpoint reagents.^45^ We also found that patients with high *ARID1A* expression, and low *IDH1* and *TP53* expression may be more sensitive to ICB therapy as judged by the TIDE score. The analysis also suggested that patients with *CIC* and *EGFR* mutations are less responsive in ICB treatment and patients without *IDH1* and *TP5*3 mutations may be more responsive to ICB therapy according to the TIDE score. ICB immunotherapy can help the immune system recognize and attack cancer cells.^21^ However, about one‐third of patients responded to ICB treatment in most types of cancers.^46^ Our study may indicate the characteristics of gliomas that facilitate the response to ICB therapy. Although experimental work is needed to identify specific mechanisms, the present study provides some new ideas for developing anti‐tumour drugs and ICIs.

Finally, combined with both univariate and multivariate Cox regression analyses, we found only *CIC* was significantly associated with both OS and PFS for patients with LGG. We identified mutated gene *CIC* as a potential biomarker in LGG and constructed a clinically dependable predictive method for predicting the survival probability of 1‐, 3‐ and 5‐year OS and PFS for patients with LGG. The predictive model showed that *CIC* can serve as potential independent biomarker in LGG.

In conclusion, we comprehensively analysed mutation profiles based on the RNA‐seq data with 510 LGG samples from TCGA database, and evaluated the prognostic value and immune infiltration of mutated genes in LGG. Our results showed that a total of 485 (95.47%) of 508 samples in LGG were altered, and Kaplan‐Meier analysis showed that 6 mutated genes were significantly correlated with OS in mutation and wildtypes. Immune infiltration analysis revealed that mutated genes were significantly involved in tumour immune microenvironment, and *CIC* can serve as a potential independent indicator for survival in LGG based on nomogram predictive model. However, these results in our present study need to be further validated in patients with LGG receiving immunotherapy in the future, and further investigation into the molecular functions of mutated genes may facilitate a better understanding on more therapeutic targets and more efficient treatment strategies against LGG.

## CONFLICT OF INTERESTS

The authors declare that the research was conducted in the absence of any commercial or financial relationships that could be construed as a potential conflict of interest. The authors confirm that there are no conflicts of interest.

## AUTHOR CONTRIBUTION

**Wen‐wen Lin:** Data curation (equal); Formal analysis (equal); Writing‐original draft (equal). **Guan‐yong Ou:** Data curation (equal); Formal analysis (equal); Writing‐original draft (equal). **Wei‐jiang Zhao:** Writing‐review & editing (equal).

## Supporting information

Figure S1‐S7

Table S1‐S2

## Data Availability

The datasets for this study can be found in the Cancer Genome Atlas (TCGA) database [http://cancergenome.nih.gov/abouttcga].
