# Supplementary material for: Mutational profiling of low‐grade gliomas identifies prognosis and immunotherapy‐related biomarkers and tumour immune microenvironment characteristics
Source: J Cell Mol Med. 2021 Oct 1;25(21):10111–25. doi: 10.1111/jcmm.16947 (PMC8572778; doi:10.1111/jcmm.16947)
Supplement: Supplementary file 1 — Figure S1‐S7 [file JCMM-25-10111-s001.docx]

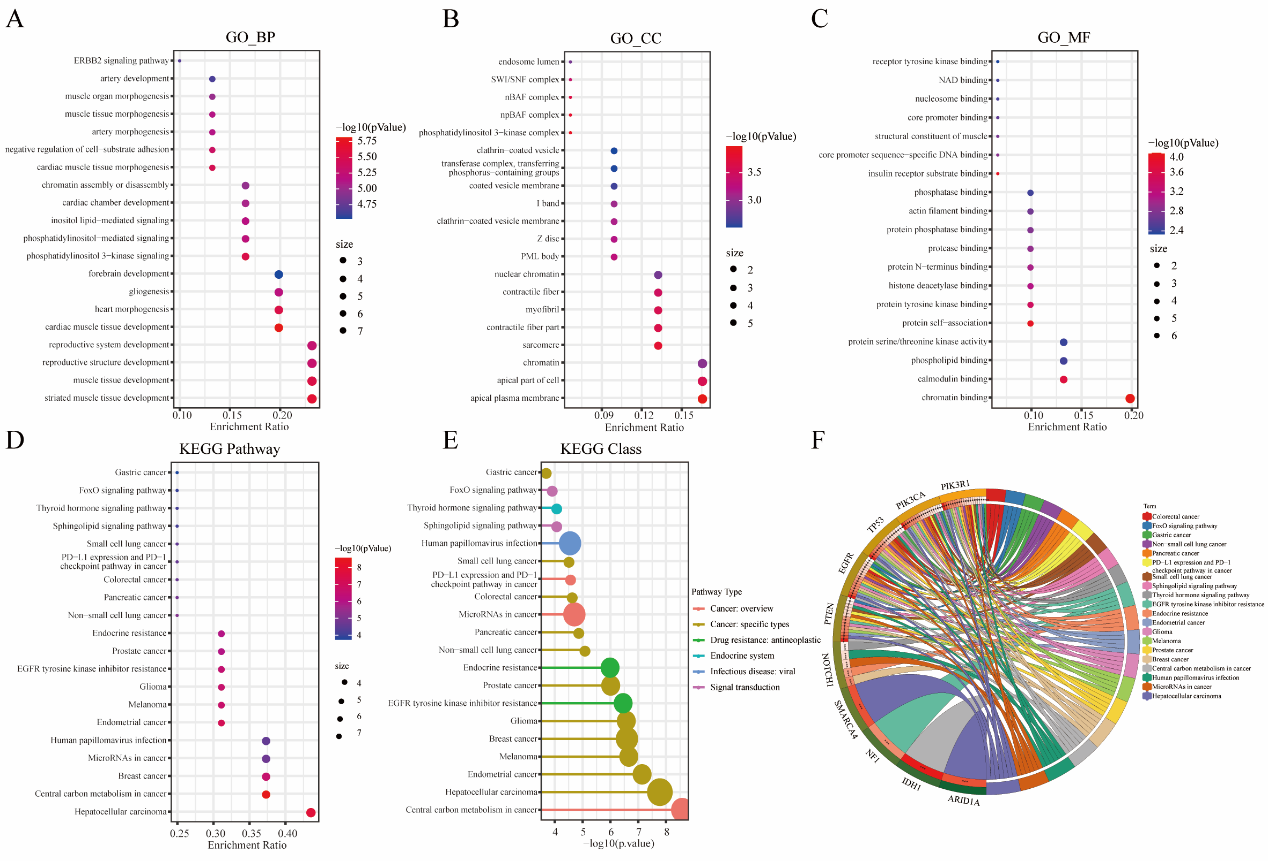


**Figure S1. Functional enrichment analysis of top 30 mutated genes.** Enriched GO BP(A), CC (B), MF (C), and KEGG pathways(D) are shown in bubble plot. Lollipop plot displays the KEGG pathway class (E), and chord shows the correlation between genes and KEGG pathways (F).


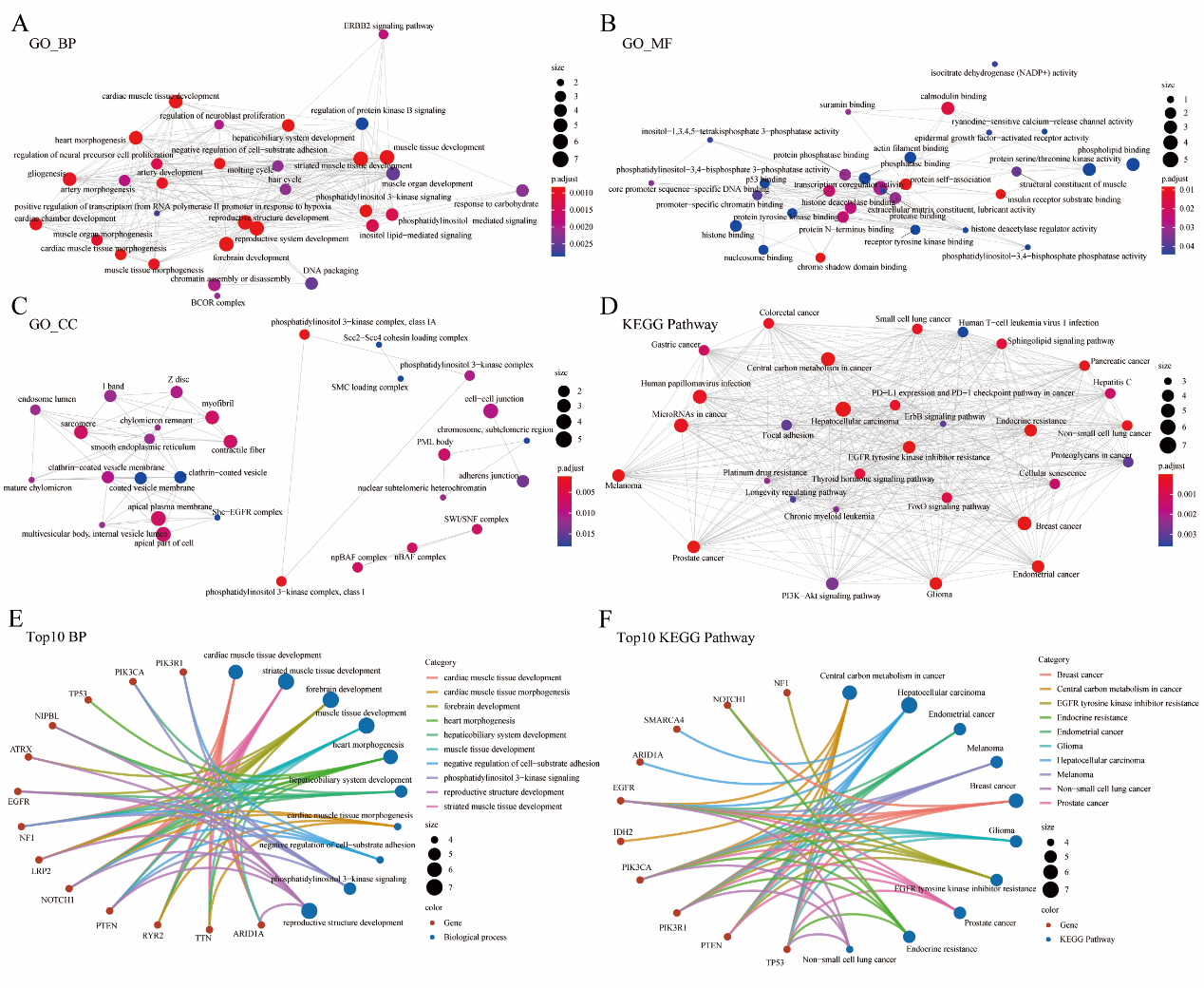


**Figure S2. GO term and KEGG pathway enrichment network analysis.** Bubble network plots show the association of different GO BP (A), MF (B), CC (C), and KEGG pathways (D). Dot-line network plots display the relationship of genes with top 10 enriched biological processes (E) and KEGG pathways (F).


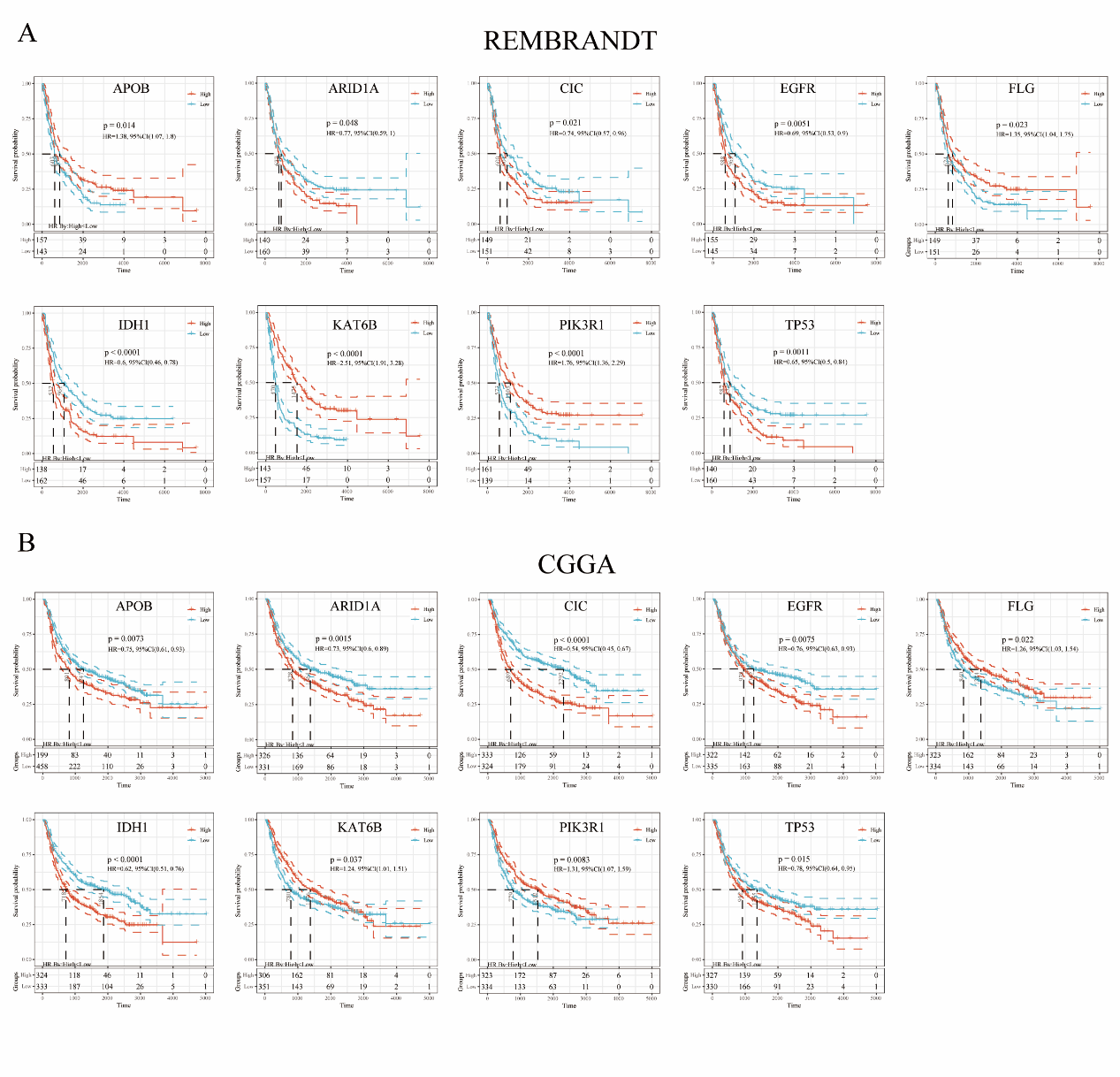


**Figure S3. Survival analysis of the screened survival-related mutated genes in the REMBRANDT (A) and CGGA database (B).**


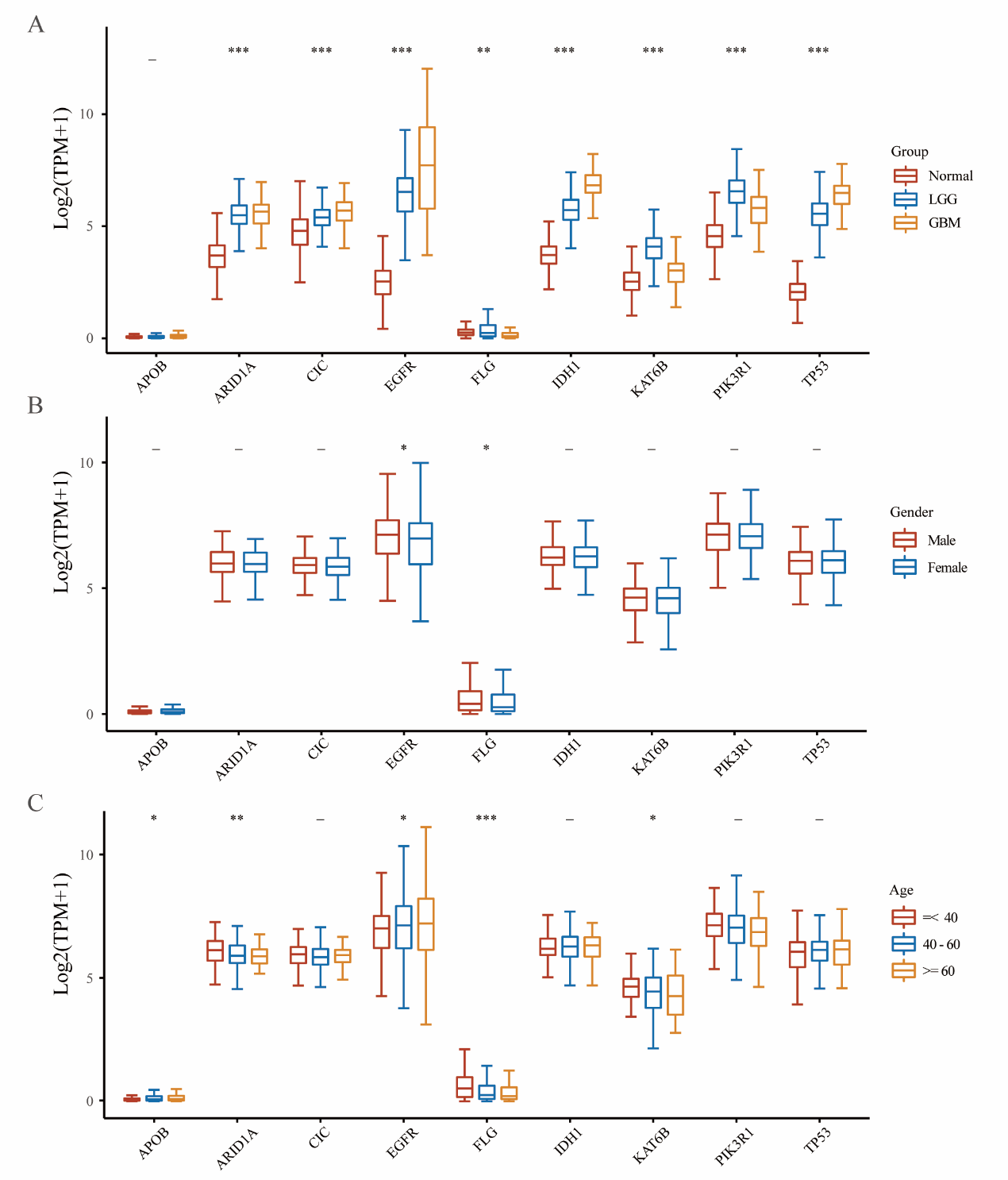


**Figure S4. The expression distribution of nine mutated genes.** The expression difference of survival-related genes in tumor tissues and normal tissue (A). The expression difference of survival-related genes on genders (B) and ages (C) in patients with LGG. The significance of two groups of samples passed the Kruskal-Wallis test. ns, no significance; * P<0.05; **, P <0.01; ***, P < 0.001


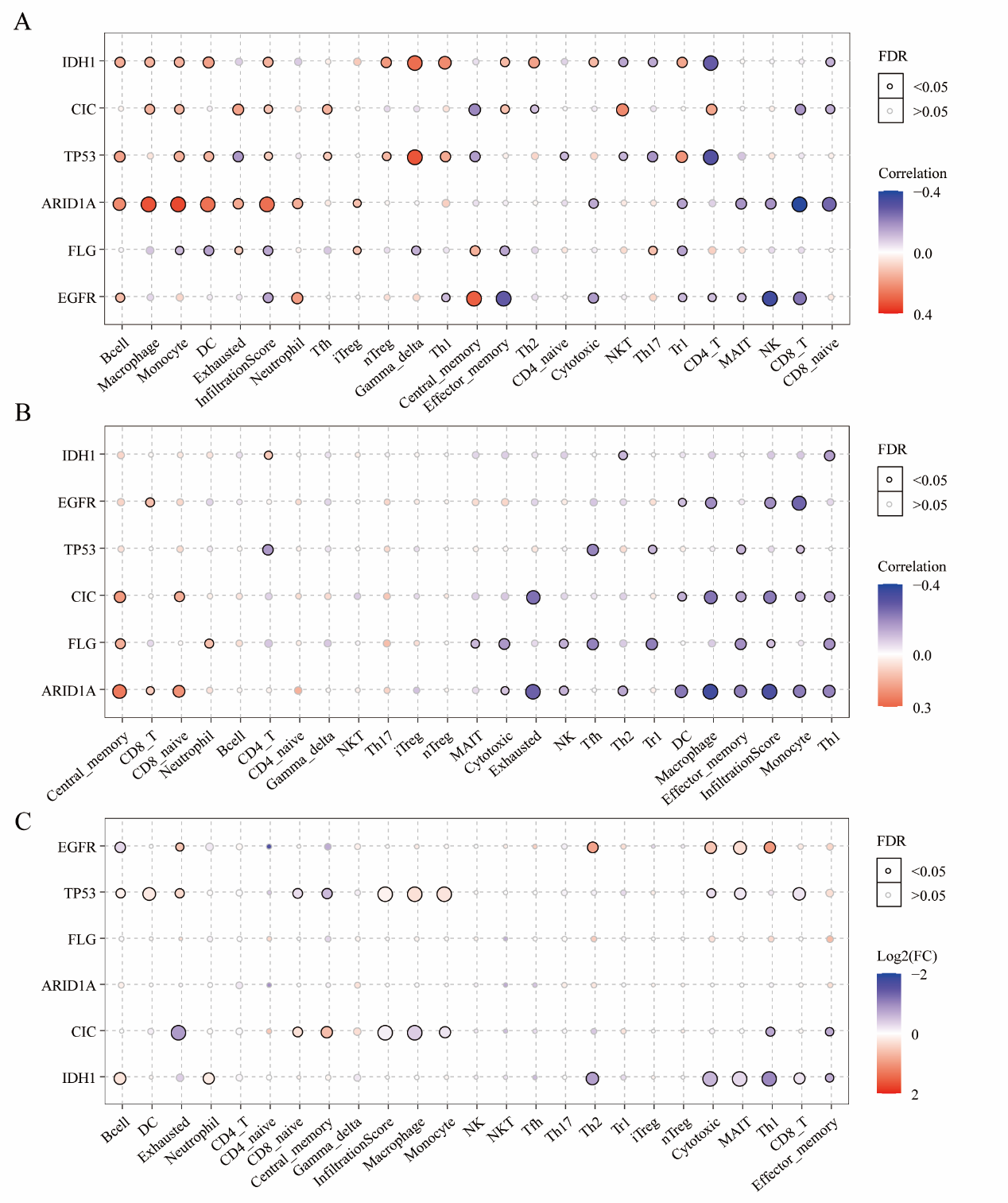


**Figure S5. Immune infiltrates analysis.** Expression correlation between six mutated genes and immune infiltrates in LGG (A). Correlation between six mutated genes methylation and immune infiltrates in LGG (B). Difference of immune infiltrates between SNV and wildtype of six mutated genes in LGG (C).


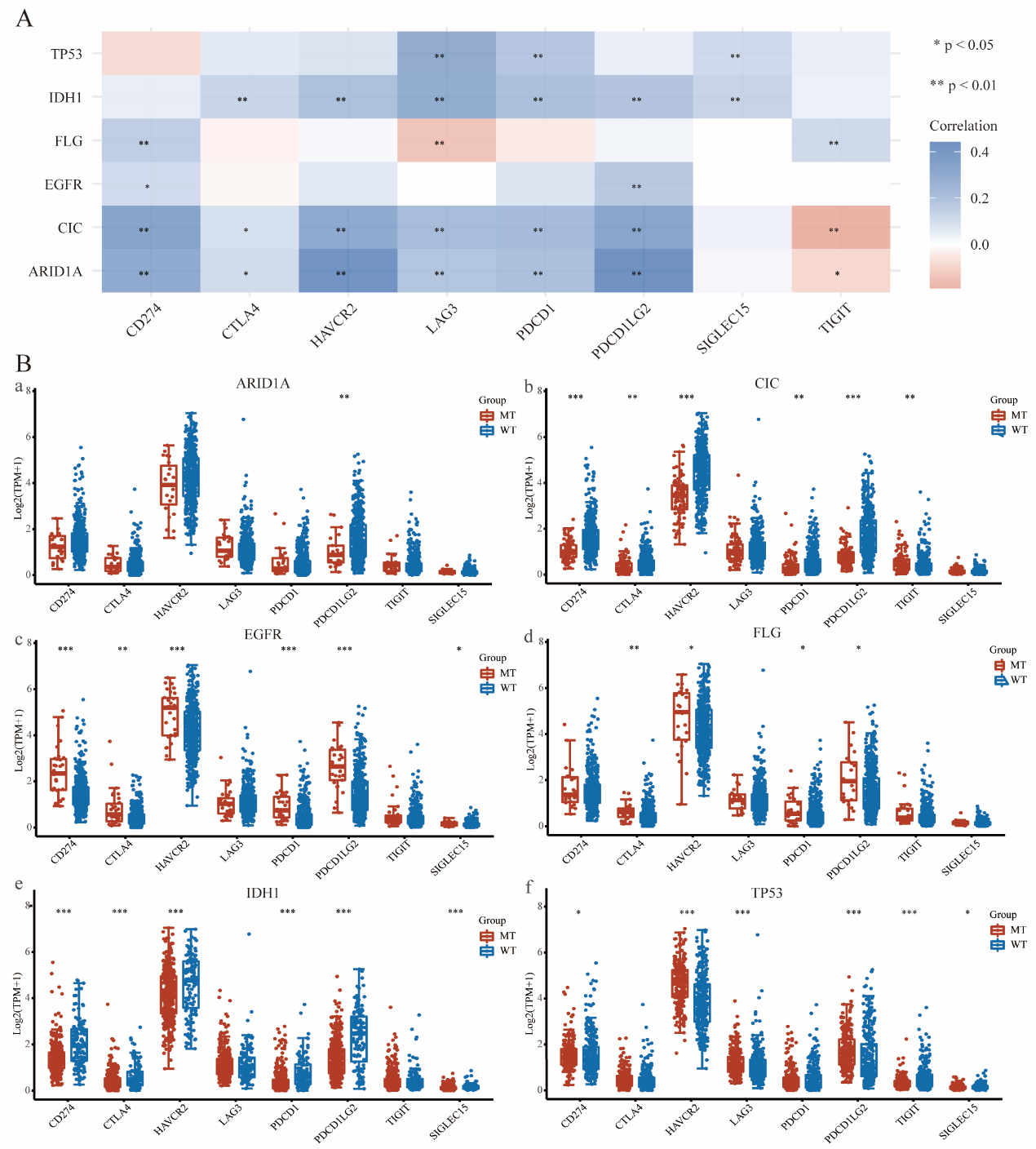


**Figure S6. Immune checkpoints analysis.** Correlation between six hub mutated gene expression and immune checkpoints related gene expression in LGG (A). The expression distribution of immune checkpoints-related genes in mutant and WT in LGG (B). MT: mutant type; WT: wildtype. The significance of the different groups of samples passed the Kruskal-Wallis test, and Spearman’s correlation analysis was used to describe the correlation. *, P < 0.05; **, P <0.01; ***, P < 0.001.


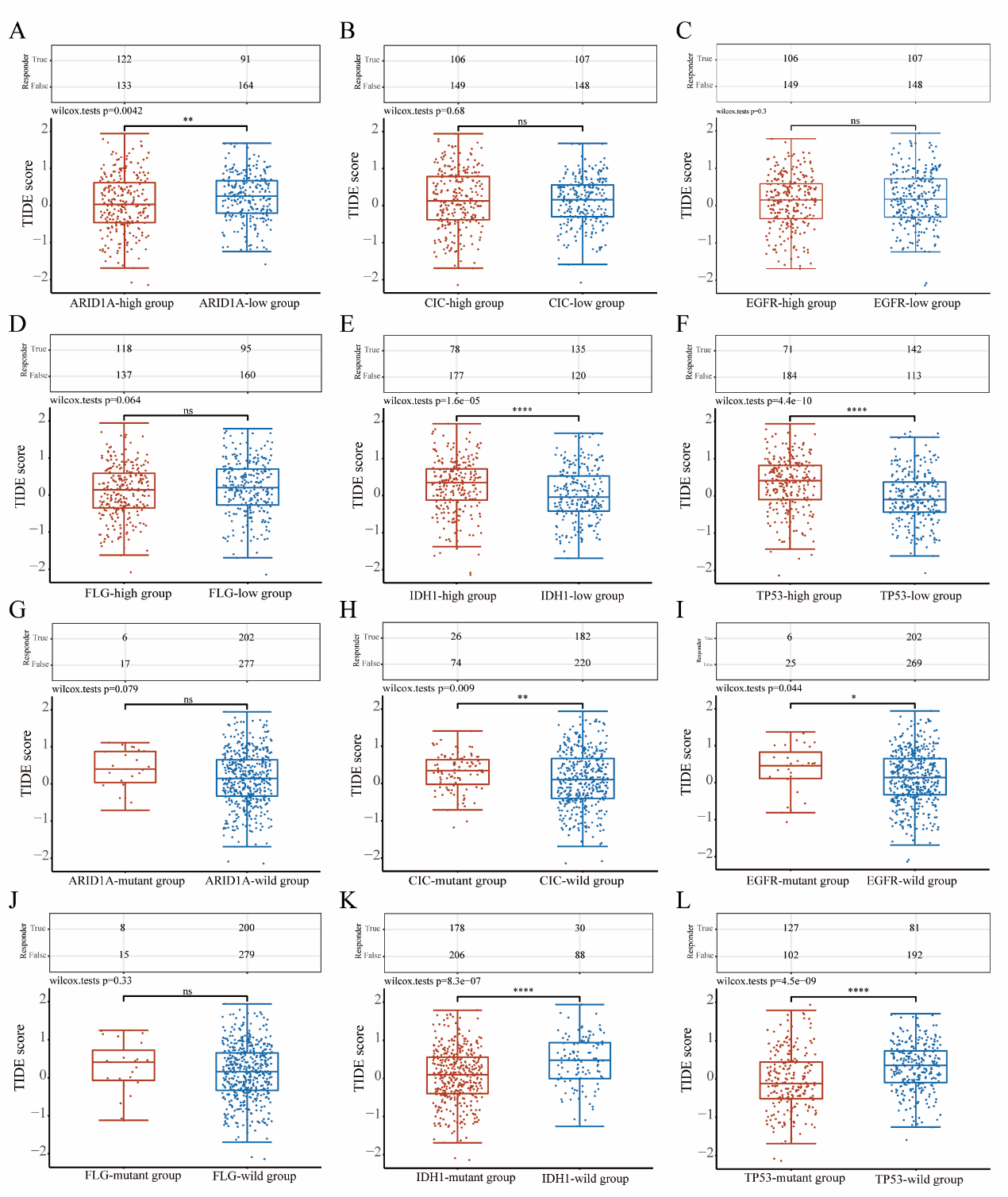


**Figure S7. TIDE signatures predict ICB immunotherapy response.** TIDE score in high- and low-expression groups of six hub mutated genes including *ARID1A* (A), *CIC* (B), *EGFR* (C), *FLG* (D), *IDH1* (E), and *TP53* (F). TIDE score in mutant- and wild groups of six hub mutated genes including *ARID1A* (G), *CIC* (H), *EGFR* (I), *FLG* (J), *IDH1* (K), and *TP53* (L). The significance of two groups of samples passed the Wilcox. test, ns, no significance; *, P < 0.05; **, P <0.01; ****, P < 0.0001.
