# Supplementary material for: Mutational profiling of low‐grade gliomas identifies prognosis and immunotherapy‐related biomarkers and tumour immune microenvironment characteristics
Source: J Cell Mol Med. 2021 Oct 1;25(21):10111–25. doi: 10.1111/jcmm.16947 (PMC8572778; doi:10.1111/jcmm.16947)
Supplement: Supplementary file 2 — Table S1‐S2 [file JCMM-25-10111-s002.docx]

**Table S1. Clinical characteristics of patients in TCGA-LGG dataset.**

|  | Characters | LGG |
| --- | --- | --- |
| Status | Alive | 385 |
|  | Dead | 125 |
| Age | Mean (SD) | 42.9(13.4) |
|  | Median [Min, Max] | 41[14, 87] |
| Gender | Female | 228 |
|  | Male | 282 |
| Race | American Indian | 1 |
|  | Asian | 8 |
|  | Black | 21 |
|  | White | 470 |
| Grade | Discrepancy | 1 |
|  | G2 | 248 |
|  | G3 | 261 |
| Radiation therapy | Non-radiation | 120 |
|  | Radiation | 142 |
| History of neoadjuvant treatment | Neoadjuvant | 1 |
|  | No Neoadjuvant | 507 |
|  | Yes, Pharmaceutical Treatment Prior to Resection | 1 |
|  | Yes, Radiation Prior to Resection | 1 |

**Table S2. Univariate and multivariate Cox regression analysis of 5 genes related to prognosis.**

|  | Variables | Univariate analysis | | | Multivariate analysis | | |
| --- | --- | --- | --- | --- | --- | --- | --- |
|  |  | HR | 95%CI | p value | HR | 95%CI | p value |
| OS | *CIC* | 1.903 | 1.329-2.723 | 0.0004 | 1.669 | 1.175-2.372 | 0.004 |
|  | *EGFR* | 1.329 | 1.170-1.511 | 1e−05 | 1.115 | 0.998-1.247 | 0.054 |
|  | *FLG* | 0.697 | 0.472-1.029 | 0.069 | 0.692 | 0.490-0.978 | 0.037 |
|  | *IDH1* | 1.751 | 1.319-2.325 | 0.0001 | 1.282 | 0.917-1.794 | 0.147 |
|  | *TP53* | 1.509 | 1.197-1.905 | 0.0005 | 1.059 | 0.807-1.389 | 0.681 |
|  | Age | 1.058 | 1.043-1.073 | < 0.0001 | 1.054 | 1.038-1.069 | <0.0001 |
|  | Gender | 1.094 | 0.767-1.561 | 0.619 | 1.138 | 0.790-1.638 | 0.488 |
|  | Grade | 3.397 | 2.296-5.024 | < 0.0001 | 2.423 | 1.601-3.667 | 3e−05 |
| PFS | *CIC* | 1.684 | 1.255-2.259 | 0.0005 | 1.56 | 1.172-2.102 | 0.0025 |
|  | *EGFR* | 1.205 | 1.088-1.335 | 0.0003 | 1.059 | 0.957-1.173 | 0.266 |
|  | *FLG* | 0.829 | 0.611-1.128 | 0.234 | 0.829 | 0.620-1.110 | 0.209 |
|  | *IDH1* | 1.446 | 1.158-1.804 | 0.001 | 1.27 | 0.969-1.681 | 0.083 |
|  | *TP53* | 1.238 | 1.039-1.475 | 0.017 | 0.956 | 0.773-1.183 | 0.680 |
|  | Age | 1.029 | 1.018-1.040 | <0.0001 | 1.027 | 1.015-1.039 | 0.00001 |
|  | Gender | 0.876 | 0.658-1.164 | 0.361 | 0.879 | 0.659-1.174 | 0.382 |
|  | Grade | 1.688 | 1.262-2.258 | 0.0004 | 1.386 | 1.021-1.882 | 0.037 |
